# Protective Efficacy of Lectin-Fc(IgG) Fusion Proteins In Vitro and in a Pulmonary Aspergillosis In Vivo Model

**DOI:** 10.3390/jof6040250

**Published:** 2020-10-27

**Authors:** Claudia Rodriguez-de la Noval, Susana Ruiz Mendoza, Diego de Souza Gonçalves, Marina da Silva Ferreira, Leandro Honorato, José Mauro Peralta, Leonardo Nimrichter, Allan J. Guimarães

**Affiliations:** 1 Laboratório de Bioquímica e Imunologia das Micoses, Departamento de Microbiologia e Parasitologia, Instituto Biomédico, Universidade Federal Fluminense, Niterói 24020-141, RJ, Brazil; claudia.noval90@gmail.com (C.R.-d.l.N.); susaruizmendoza@gmail.com (S.R.M.); fusariumsp@gmail.com (D.d.S.G.); marinaferreira83@gmail.com (M.d.S.F.); 2 Laboratório de Glicobiologia de Eucariotos, Instituto de Microbiologia Professor Paulo de Góes, Universidade Federal do Rio de Janeiro, Rio de Janeiro 21941-902, RJ, Brazil; lhonoratobr@gmail.com (L.H.); nimrichter@micro.ufrj.br (L.N.); 3 Departamento de Imunologia, Instituto de Microbiologia Paulo de Góes, Universidade Federal do Rio de Janeiro, Rio de Janeiro 21941-902, RJ, Brazil; peralta@micro.ufrj.br; 4 Pós-Graduação em Doenças Infecciosas e Parasitárias, Faculdade de Medicina, Universidade Federal do Rio de Janeiro, Rio de Janeiro 21941-913, RJ, Brazil; 5 Programa de Pós-Graduação em Microbiologia e Parasitologia Aplicadas (PPGMPA), Instituto Biomédico, Universidade Federal Fluminense, Rua Professor Hernani Pires de Melo 101, São Domingos, Niterói 24210-130, RJ, Brazil

**Keywords:** Fc-fusion proteins, passive immunization, *Aspergillus fumigatus*, pathogenesis

## Abstract

Aspergillosis cases by *Aspergillus fumigatus* have increased, along with fungal resistance to antifungals, urging the development of new therapies. Passive immunization targeting common fungal antigens, such as chitin and β-glucans, are promising and would eliminate the need of species-level diagnosis, thereby expediting the therapeutic intervention. However, these polysaccharides are poorly immunogenic. To overcome this drawback, we developed the lectin-Fc(IgG) fusion proteins, Dectin1-Fc(IgG2a), Dectin1-Fc(IgG2b) and wheat germ agglutinin (WGA)-Fc(IgG2a), based on their affinity to β-1,3-glucan and chitooligomers, respectively. The WGA-Fc(IgG2a) previously demonstrated antifungal activity against *Histoplasma capsulatum*, *Cryptococcus neoformans* and *Candida albicans*. In the present work, we evaluated the antifungal properties of these lectin-Fc(s) against *A. fumigatus*. Lectin-Fc(IgG)(s) bound in a dose-dependent manner to germinating conidia and this binding increased upon conidia germination. Both lectin-Fc(IgG)(s) displayed in vitro antifungal effects, such as inhibition of conidia germination, a reduced length of germ tubes and a diminished biofilm formation. Lectin-Fc(IgG)(s) also enhanced complement deposition on conidia and macrophage effector functions, such as increased phagocytosis and killing of fungi. Finally, administration of the Dectin-1-Fc(IgG2b) and WGA-Fc(IgG2a) protected mice infected with *A. fumigatus*, with a 20% survival and a doubled life-span of the infected mice, which was correlated to a fungal burden reduction in lungs and brains of treated animals. These results confirm the potential of lectin-Fc(IgGs)(s) as a broad-spectrum antifungal therapeutic.

## 1. Introduction

Invasive fungal infections, such as aspergillosis caused by *Aspergillus fumigatus,* are a growing public health problem with high levels of morbidity and mortality in many countries [1]. Humans are exposed to large numbers of *Aspergillus’* conidia on daily base [2], and immunocompromised patients, including elderly and premature newborn, patients with AIDS [3], as well those under immunosuppressive therapy or undergoing solid cell or hematopoietic cell transplantation [4,5], suffering from hematologic cancer [6], neutropenia or malnutrition [7,8], can develop invasive aspergillosis [9]. *A. fumigatus* and *A. flavus* are the *Aspergillus* species with medical importance causing death in immunocompromised patients [10]. In particular, *A. fumigatus* is responsible for the majority of allergic fungal disease [11,12,13], with ~3,000,000 cases of chronic pulmonary aspergillosis and 250,000 of invasive aspergillosis (IA) annually [12].

Aspergillosis is generally difficult to diagnose, with an often complicated and ineffective treatment, resulting in a high mortality rate (10–40%) [14]. Triazoles such as itraconazole, voriconazole, and posaconazole are the main oral therapy for *A. fumigatus* infections [15]. Intravenous amphotericin B (AMB) is also authorized for the treatment of invasive aspergillosis; however, its use is limited to chronic and allergic aspergillosis due to adverse effects and high toxicity [16,17]. Echinocandins might be useful when the other drugs are contraindicated [18]. The emergence of *Aspergillus* spp. antifungal resistance and the appearance of new multi-resistance are additional factors that contribute to the decrease of intervention options and the absence of an effective therapeutic strategy against *A. fumigatus* [19]. Therefore, the development of new strategies becomes necessary [17,20].

Although vaccines have been extensively explored in murine models [21,22], their use is sometimes limited, especially when the major target patients are immunocompromised and lack efficient adaptive immunity [23]. Passive immunization using immune serum or monoclonal antibodies (mAbs), however, offers an alternative and promising option to this group of patients [24]. MAbs and antigen-binding fragment (Fab) have already demonstrated efficacy against *Cryptococcus neoformans* [25,26], *Candida albicans* [27,28], *A. fumigatus* [26,29,30] and *Histoplasma capsulatum* [31,32,33,34].

A desirable feature is the use of mAbs recognizing antigens that are shared by different fungal species, to target as many strains as possible [35] and thereby eliminating the need for species-level diagnosis, a complicated and time-consuming task. Ubiquitous antigens such as β-glucans and chitin [23] are important elements of the cell wall structure of several pathogenic fungal species and confer integrity, rigidity, and resistance to the extracellular environment [36,37,38,39]. Therefore, their use as targets would enable the development of a broad-spectrum passive immunization therapy and boost the development of antifungal immunotherapeutic strategies.

However, most fungal carbohydrates, are T-cell independent antigens with low antigenicity, and frequently induce low antibody titers, which impairs mAbs production. An interesting alternative is the construction of fusion proteins composed of lectins, natural carbohydrate ligands, directly linked to the Fc domain of an immunoglobulin (Ig), replacing its binding arms and promoting specific recognition of fungal antigens. Wheat germ agglutinin (WGA), for example, has affinity for N-acetylglucosamine oligomers and can recognize chitin [40]; whereas Dectin-1, a C-type lectin, is considered the largest non-opsonic β-1,3-glucan receptor [41]. Besides, the presence of an Fc region could also improve solubility, stability and facilitate the purification process [42]. Based on these concepts, three chimeric proteins containing the polysaccharide-binding site of these lectins fused with murine IgG Fc portions were recently constructed by our group [43,44]. The WGA-Fc(IgG2a) antifungal functions were characterized against the yeasts *C. albicans*, *C. neoformans* and *H. capsulatum*, and the recombinant proteins were shown to directly inhibit fungal growth and to interfere with morphogenesis [43,44]. In addition, the Fc portion also promotes antifungal mechanisms of immunity (innate and cellular) by conferring immunoglobulin effector functions, such as activating complement deposition and due to Fc-receptors engagement, increasing phagocytosis and killing by phagocytic cells. Moreover, WGA-Fc(IgG2a) was also shown to provide in vivo antifungal efficacy [44].

In this work, taking advantage/benefitting of the existing of WGA-Fc(IgG2a) with potential antifungal functions and two comparable Fc-fusion proteins produced in our laboratory, Dectin-1-Fc(IgG2a) and Dectin-1-Fc(IgG2b), we demonstrate that they efficiently recognized *A. fumigatus* germinating conidia, and were able to increase complement activation, phagocytosis by macrophages and their antifungal functions. Co-incubation with these Fc-fusion proteins also delays hyphae elongation, and their prophylactic administration was able to increase the life-span of infected mice and thereby to protect them against *A. fumigatus* infection, resulting in a greatly reduced pulmonary fungal burden. Therefore, we suggest that these lectin-Fc(IgG) proteins are potential immunobiologicals with a broad-spectrum strategy, including the management of aspergillosis.

## 2. Materials and Methods

### 2.1. Animal Use and Ethics Statement

Male C57BL/6 mice (6–8 weeks old) were obtained from the Laboratory Animal Center (NAL) of the Fluminense Federal University (Niteroi, RJ, Brazil). The animals were housed in specific pathogen-free facilities and handled according to institutionally recommended guidelines. All in vivo experiments were performed accordingly to approved protocols by the Ethics committee for animal use (CEUA) of the Fluminense Federal University (Protocols 600/2014 and 5487190618/2018).

### 2.2. Organisms and Growth Conditions

*A. fumigatus* NCPF 2109 (ATCC^®^ 46645 TM) strain was grown on Potato Dextrose Agar (PDA; Neogen, Lansing, MI, USA) supplemented with 5% penicillin-streptomycin (Gibco^®^-Life Technologies) and isolation of conidia was performed as described, with some slight modifications [45,46,47]. The cultures were maintained at 37 °C for 5 to 7 days and conidial suspensions were prepared by extensively washing the Petri dishes with 10 mL of sterile 1× PBS solution (phosphate buffered saline, 137 mM NaCl, 2.7 mM KCl, 1.5 mM KH_2_PO_4_, 8.1 mM Na_2_HPO_4_, pH 7.4) containing 0.1% Tween 20. The hyphal fragments were removed using a cell strainer (40 µm, Falcon; Whitley Bay, UK) and the viable resting conidia were diluted in PBS or Dulbecco’s Modified Eagle Medium (DMEM) and enumerated using a hemocytometer. When fixed resting conidia (0 h) were needed, 4% paraformaldehyde in PBS was added. The conidia were incubated for 20 min and finally washed with PBS.

### 2.3. Bone Marrow-Derived Macrophages Isolation

Bone marrow-derived macrophages were obtained from 6–8 weeks C57BL/6 mice and cultured in DMEM medium (Gibco^®^-Life Technologies; Waltham, MA, USA) supplemented with 10% Fetal Bovine Serum (FBS, Cultilab, Campinas, Brazil), 1% nonessential amino acids and 1% penicillin-streptomycin (Gibco^®^-Life Technologies; Waltham, MA, USA). The medium was also supplemented with 15% L929 cell conditioned medium, as a source of granulocyte/macrophage colony stimulating factor (GM-CSF), to differentiate macrophages [48,49], and the cells were kept at 37 °C in a 5% CO_2_ atmosphere. After a week, macrophages were recovered and used in all experiments.

### 2.4. Construction of Lectin-Fc(IgG) Fusion Proteins

RNAs were extracted from mouse macrophages or *Triticum aestivum* following the standard TRIzol^®^LS protocol and cDNAs was produced according to the SuperScript III First-Strand Synthesis System (ThermoFisher Scientific, Waltham, MA, USA). The mouse cDNA was used to amplify the Dectin-1 coding sequence (CLEC.7, GenBank BC027742.1) using the oligonucleotide pair Dectin-1 forward and Dectin-1 reverse (Table 1), whereas the *T. aestivum* cDNA was used to amplify the WGA sequence (GenBank M25536.1) with the oligonucleotide pair WGA forward and WGA reverse (Table 1). PCR amplification was set up according to Liedke et al. [44], with exception of the annealing temperatures of 62 °C for Dectin-1 and 56 °C for WGA. Dectin-1 and WGA amplicons were digested with endonucleases pair *EcoR*I/*Bgl*II and *EcoR*I/NcoI, respectively (ThermoFisher Scientific, Waltham, MA, USA). Plasmids from the pFUSE-mIgG-Fc(IgG2a or IgG2b) family, were digested with the respective pairs of endonucleases and used in ligation reactions for cloning of the respective lectins, to construct the plasmids with WGA lectin fused to the Fc region of IgG2a, or Dectin-1 fused to the effector region of IgG2a and IgG2b, respectively [43,44]. Plasmids integrity was confirmed by PCR as above, endonuclease digestion and sequencing. Transfections to CHO-k1 cells were performed as described [44].

### 2.5. Lectin-Fc(IgG) Fusion Proteins Expression and Purification

Expression of lectin-Fc(IgG) proteins Dectin-1-Fc(IgG2a), Dectin-1-Fc(IgG2b) and WGA-Fc(IgG2a) by CHO-k1 transfectants was performed in Ham’s F-12 (HyCloneTM-GE Healthcare Life Sciences; Marlborough, MA, USA) supplemented with 1.2 g/L NaHCO_3_, 10% (*v*/*v*) fetal bovine serum (FBS)(Cultilab, Campinas, Brazil), 1% (*v*/*v*) nonessential amino acids (Gibco^®^-Life Technologies, Waltham, MA, USA), 1% penicillin/streptomycin (Gibco^®^-Life Technologies, Waltham, MA, USA), Zeocin (70 µg/mL), 1.5 g/L yeast extract and 1.5 g/L peptone (Kasvi; São José dos Pinhais, Paraná, Brazil). The cells were grown at 37 °C in a 5% CO_2_ atmosphere and the supernatants were collected 7 days after they reached 100% confluence. Lectin-Fc(IgG) fusion protein expression was confirmed by indirect enzyme-linked immunosorbent assay (ELISA) using a Nunc maxisorp 96-well microplate (ThermoScientific, Waltham, MA, USA) pre-coated with chitin or laminarin, respectively [44]. The collected supernatant was centrifuged for 10 min at 10,000× *g* and filtered using a 0.22 μm membrane to remove cellular debris. Purification of lectin-Fc(IgG) fusion proteins was performed with a HiTrap Protein A HP column according to manufacturer’s instructions (GE Healthcare Life Sciences, Singapore).

### 2.6. Molecular Characterization of Lectin-Fc(IgG) Proteins

The molecular weight of the purified lectin-Fc(IgG) proteins was evaluated by Western blot. First, the proteins were separated by SDS-PAGE (Sodium Dodecyl Sulfate Polyacrylamide Gel Electrophoresis) [37,50]. Previously, approximately 1.0 μg of each protein were diluted in non-reducing (Tris-HCl 12.5 mM pH 6.8, 4% glycerol, 0.4% SDS and 0.005% bromophenol blue) and reducing buffer (as above, but adding 1% ß-mercaptoethanol), and were incubated at 95 °C for 5 min. After the electrophoretic separation, the proteins were transferred to a nitrocellulose membrane (0.45 μm) (NC-Hybond—Amersham Biosciences, Little Chalfont, UK) and blocked with 5% milk solution in 0.1% TBS-T (Tris buffered saline, 20 mM Tris, 150 mM NaCl, 0.1% Tween 20, pH 7.5) for 1 h. A goat anti-mouse IgG conjugated with alkaline-phosphatase (AP) was used for immunodetection. The membrane was washed three times (3×) with TBS-T buffer for 10 min after each incubation and finally developed using the nitro blue tetrazolium (NBT)/5-bromo-4-chloro-3-indolyl-phosphate (BCIP) (ThermoScientific, Waltham, MA, USA) substrate until visualization of bands.

### 2.7. Binding of Lectin-Fc(IgG) Proteins to A. fumigatus Conidia

An indirect ELISA was used to verify the binding of the fusion proteins to the cell wall structures of *A. fumigatus*. The fungal cells after 2 h of germination were fixed with 4% paraformaldehyde at room temperature (RT). Further germination resulted in germ tube and hyphae formation that reduced adherence to ELISA plates. First, Nunc maxisorp 96-well plates were coated with 10^6^ conidia/well in PBS and incubated at 37 °C for 1 h. Plates were blocked with 1% bovine serum albumin (BSA) diluted in TBS-T and then incubated at 37 °C for 1 h with a serial dilution (1:2) of each lectin-Fc(IgG) protein, starting at a concentration of 10 μg/mL. After a 1 h incubation, a solution of anti-mouse IgG alkaline-phosphatase conjugated (1 μg/mL) (Southern Biotech; Birmingham, AL, USA), diluted in blocking solution was added and plates incubated again at 37 °C for 1 h. The plates were washed 3× with TBS-T after each incubation. Finally, the plates were incubated with 1 mg/mL p–nitrophenyl phosphate substrate (pNPP; Sigma Aldrich; St. Louis, MO, USA) at RT for 30 min and read at 405 nm in a SpectraMax 190 microplate reader (Molecular Devices; San Jose, CA, USA).

An inhibition ELISA was also performed as described elsewhere [44,51], by initially coating Nunc maxisorp “reaction” plates with 10 μg/mL of either laminarin or chitin, for Dectin-1-Fc(IgG) or WGA-Fc(IgG2a), respectively. The “reaction” plates were incubated for 1 h at 37 °C, followed by an overnight step at 4 °C. A separate “inhibition” plate was blocked with 1% BSA in TBS-T for 1 h at 37 °C and serial dilutions (1:3) of *A. fumigatus* germinating conidia were added (starting at 4 × 10^7^ cells/well). Upon addition of each respective lectin-Fc(IgG) with a final concentration of 5 μg/mL, inhibition plates were incubated for 1 h at 37 °C. The contents of the “inhibition” plate were transferred to the “reaction” plate that was previously blocked with 1% BSA in TBS-T and plate was incubated again at 37 °C for 1 h. Incubation with anti-mouse IgG alkaline-phosphatase conjugate (1 μg/mL), washing steps after each incubation and development with pNPP substrate solution (1 mg/mL) were performed as described above. Finally, the plates were read in a SpectraMax microplate reader at 405 nm.

### 2.8. Conidia Germination and Lectin-Fc(IgG) Labeling by Immunofluorescence

The binding patterns of the lectin-Fc(IgG) to the fungal cell wall were also evaluated by immunofluorescence [31]. First, *A. fumigatus* conidiophores were isolated from PDA plates as described. *A. fumigatus* resting conidia were fixed as described (0 h) or incubated for 2, 4 or 8 h at 37 °C with RPMI-1640 medium supplemented with 5% FBS (Cultilab, Campinas, Brazil) for germination and then fixed in 4% paraformaldehyde. Then, 5 × 10^6^ resting, germinating conidia (2, 4 or 8 h) or conidiophores were incubated for 1 h at RT with 2 μg/mL of either Dectin-1-Fc(IgG2a), Dectin-1-Fc(IgG2b) or WGA-Fc(IgG2a). WGA protein conjugated to TRITC (Tetramethylrhodamine) (Sigma-Aldrich) and Dectin-1 biotinylated/ streptavidin-Alexa 488 conjugate were used as controls. Cells were further incubated with a 1 μg/mL solution of a goat anti-mouse IgG-Alexa 488 (LifeTechnologies, Singapore) for 1 h at RT. Finally, the conidia were incubated in 100 μL of a solution of 1 mg/mL Uvitex 2B (Polysciences, Inc., Warrington, PA, USA) in TBS-T at RT for 15 min to highlight the chitin present on the fungal cell wall. Cells were washed 3× with PBS after each incubation. Finally, lectin-Fc(IgG) labeling of conidia was examined by flow cytometry with a FACSCalibur TM (BD Bioscience, Franklin Lakes, NJ, USA). The percentage of fluorescent cells and FL1 intensity were analyzed using FlowJo 8.7 software (Becton Dickinson, Franklin Lakes, NJ, USA). Germinating conidia in a 24-well plate (TPP^®^-Merck; Kenilworth, NJ, USA) with sterile glass coverslips and conidiophores were also treated as described above and finally, the coverslips were washed and analyzed by fluorescence microscopy in the Axio Imager Microscope (Carl Zeiss MicroImaging, Inc.; Jena, Germany) using a 100× objective. Images were analyzed by Image J (NIH, Bethesda, Rockville, MD, USA) and Adobe Photoshop CS6 (Adobe Systems Software, San Jose, CA, USA).

### 2.9. Growth Inhibition Assay Using Lectin-Fc(IgG) Proteins

A. fumigatus resting conidia were isolated, as described above, and washed with sterile PBS and centrifugations at 1750× g for 15 min. Then, 104 conidia/well were plated in 96-well culture plate (TPP, Switzerland) and incubated in 100 µL of RPMI-1640 medium supplemented with 5% FBS and 1% penicillin-streptomycin and containing 10 μg/mL of either Dectin-1-Fc(IgG2a), Dectin-1-Fc(IgG2b) or WGA-Fc(IgG2a) proteins or controls of WGA, Dectin-1 or PBS. Cells were maintained at 37 °C for 0, 1, 3, 5, 7 or 9 h. Upon each incubation-time, cells were fixed as described and images were recorded using an Axio Imager Microscope equipped with a 100× objective. The percentage of hyphae formed, and their sizes were determined at each time-point using the cellSens software (Olympus Life Science, Tokyo, Japan) and the data were compared with the control groups. Approximately 500 cells were analyzed in each condition. Experiments were performed three times in duplicates.

### 2.10. Biofilm

The effect of lectin-Fc(IgG) fusion proteins on biofilm formation was also analyzed. Briefly, *A. fumigatus* conidia were washed with PBS and suspended in RPMI-1640 medium supplemented with 5% FBS [4] (Cultilab) and 1% penicillin-streptomycin (Gibco^®^-Life Technologies, Waltham, MA, USA), at a final concentration of 1 × 10^6^ conidia/mL [4,52,53,54]. Then, cells were co-incubated in 96-well plates with Dectin-1-Fc(IgG2a), Dectin-1-Fc(IgG2b) or WGA-Fc(IgG2a) at concentrations ranging from 10 to 1 μg/mL, adding up a volume of 100 μL per well. As a control for growth inhibition, PBS, amphotericin B (4 μg/mL) [55] and boiled conidia were used. Finally, the plates were incubated at 37 °C and 5% CO_2_ for 24 and 48 h [52]. To measure biomass formation, plates were treated with 1% crystal violet (Merck, Kenilworth, NJ, USA) for 20 min at RT, as described elsewhere [56]. The wells were washed 3× and incubated for 5 min with 200 μL of absolute ethanol. After incubation, the homogenate content was transferred to a new plate and the optical density of each well was measured at 570 nm using a SpectraMax 190 microplate reader.

### 2.11. Lectin-Fc(IgG) Complement Activation and A. fumigatus Growth

To evaluate the effect of lectin-Fc(IgG) on the complement activation, 5 × 10^6^ *A. fumigatus* 2 h germinating conidia were pre-fixed and washed 3× with PBS and opsonized for 1 h at 37 °C with 2 μg/mL of each lectin-Fc(IgG). Then, the cells were incubated with 10% of pre-collected mouse serum (or inactivated serum at 100 °C for 30 min), or individual controls (Dectin-1, WGA and PBS) for 1 h at RT. To analyze the binding of complement proteins to fungal cells, the conidia were incubated with 5 μg/mL FITC-conjugated goat anti-mouse C3 (MP Biomedicals, Santa Ana, CA, USA), washed 3× and analyzed by flow cytometry using a FACSCalibur. The samples were also examined by fluorescence microscopy using an Axio Imager Microscope. For microscopic analysis, fungal cells were additionally incubated with a 0.1% solution of Uvitex 2B (Polysciences, Inc., Warrington, PA, USA) for cell wall chitin staining. The assay was performed in duplicate and repeated three times independently. 

### 2.12. Interference of Lectin-Fc(IgG) with the Interaction between Fungal Cells and Macrophages

An interaction assay was performed to evaluate the influence of the lectin-Fc(IgG) proteins on the association (adhesion and internalization) of fungi by macrophages. Briefly, 8 × 10^5^ bone-marrow derived macrophages/well were plated in a 24-well cell culture plate containing DMEM supplemented with 10% FBS and 1% of penicillin/streptomycin and were incubated overnight at 37 °C in 5% CO_2_. *A. fumigatus* 2 h germinating conidia were washed 3× with PBS and labeled with 0.04 mg/mL NHS-Rhodamine (Thermo Scientific, Waltham, MA, USA) for 30 min at RT in the absence of light. The fungal cells were washed 5× with PBS until removal of the excess of NHS-Rhodamine and were incubated for 1 h at RT with 5 μg/mL of either Dectin-1-Fc(IgG2a), Dectin-1-Fc(IgG2b) or WGA-Fc(IgG2a) and controls of WGA, Dectin-1 or PBS. After the incubations, conidia were washed 3× with PBS, and added separately to the macrophages (2 conidia: 1 macrophage ratio) and co-cultured for 2 h at 37 °C in 5% CO_2_. After the interaction, the wells were washed with sterile PBS and incubated for 15 min with a 1 mg/mL solution of Uvitex 2B, for specific staining of macrophage adhered (outside) conidia. Finally, the cells were washed 3× with sterile PBS, fixed for 30 min with 4% paraformaldehyde and analyzed using a fluorescence microscope Axio Imager Microscope. Interaction rate was calculated as the number of macrophages associated with fungal cells (macrophages containing Rhodamine positive fungi) divided by the total number of macrophages. The adhesion rate was calculated as the number of macrophages with attached fungal cells (macrophages containing Uvitex positive/ Rhodamine positive fungi) divided by the total number of macrophages whereas the phagocytosis rate was determined as the ratio of macrophages with internalized fungal cells (macrophages containing Uvitex negative/ Rhodamine positive fungi) by the total number of macrophages [33,57,58]. All values were multiplied by 100 for percentage calculations.

### 2.13. Macrophage Killing/Fungal Growth Inhibition Assay

A colony forming unit (CFU) inhibition assay was performed to evaluate the influence of lectin-Fc(IgG) proteins on fungal growth in the macrophage intracellular environment. Briefly, 2 × 10^5^ bone-marrow derived macrophages/well were plated in a 96-well plate containing 200 μL of DMEM medium supplemented with 5% FBS and 1% of penicillin-streptomycin. The macrophages were maintained at 37 °C in an atmosphere of 5% CO_2_ for 24 h. *A. fumigatus* 2 h germinating conidia were washed 3× with sterile PBS and incubated for 1 h at RT with either 5 μg/mL of WGA-Fc(IgG2a), Dectin-1-Fc(IgG2a) and Dectin-1-Fc(IgG2b) proteins, WGA, Dectin-1 or PBS. Then, the cells were incubated with the macrophages (2 conidia: 1 macrophage ratio) at 37 °C in 5% CO_2_. After the first two hours of incubation, the conidia that had not yet been phagocytosed were washed away, new media was added, and the cells were incubated further for 18 h. The wells then were washed with sterile PBS and the macrophages were lysed by the addition of 100 μL of sterile distilled water. The fungal cells from each well were plated on PDA plates and incubated for 24 h at 37 °C. The CFUs were counted and the number of colonies compared among the groups [44].

### 2.14. In Vivo Models

C57BL/6 (8-12 weeks old) male mice were immunosuppressed with intraperitoneal injection with cyclophosphamide (150 mg/kg body weight) and subcutaneous injection with cortisone acetate (112 mg/kg body weight) at days -3 and -1 prior to infection and a cyclophosphamide injection 3 days after [59,60]. For survival experiments, the immunosuppressed mice were infected intratracheally with 1 × 10^6^ of resting conidia, 1 h after they received a solution containing 10 μg of either Dectin-1-Fc(IgG2b) or WGA-Fc(IgG2a), by intraperitoneal injection. The control group was injected with PBS and infected with the same conidia inoculum.

Additionally, (6–8/group) immunosuppressed animals were infected intratracheally with a sublethal inoculum of *A. fumigatus* conidia (5 × 10^5^) as described above, and mice were euthanized 4 days post-infection. Lung, spleen and brain tissue were extracted, weighted, macerated and plated on PDA plates, for determination of fungal load. The CFUs were enumerated after 24 h of incubation at 37 °C and compared to the control groups.

### 2.15. Statistical Analysis

All statistical analyses were performed using GraphPad Prism 8 for Mac (Version 8.1.0; GraphPad Software, San Diego, CA, USA). Data comparison among groups was performed by ordinary One-Way ANOVA, with multiple comparison performed by Tukey’s or Dunnett’s correction test, for comparison of every group or to control, with a single pooled variance. Survival experiments data were analyzed by the Mantel-Cox test to determine the differences among groups.

## 3. Results

### 3.1. Construction, Expression and Molecular Characterization of Lectin-Fc(IgG) Proteins

**Figure 1:**
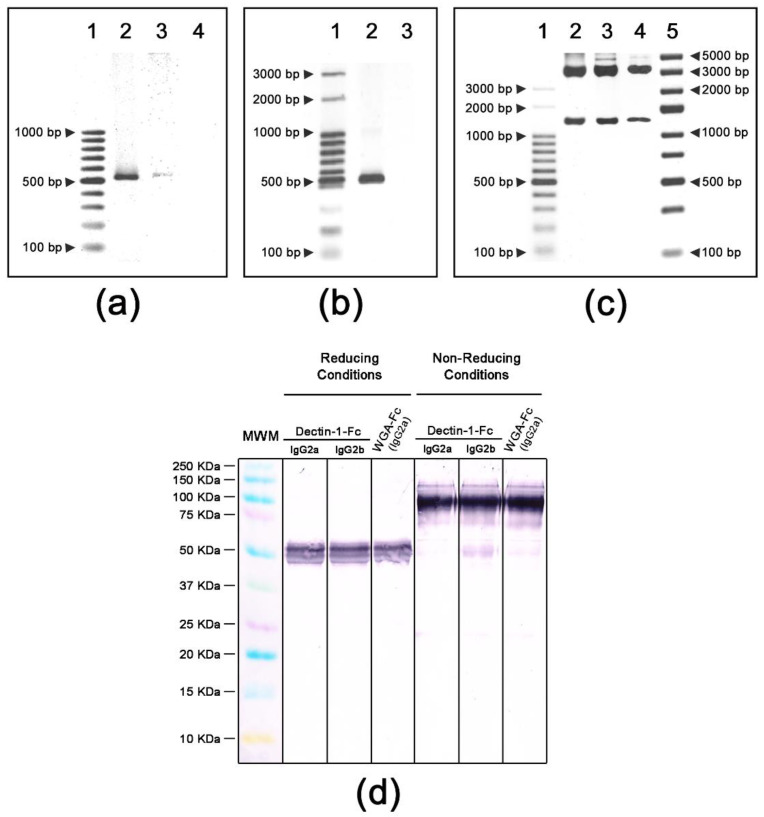
Construction of lectin-Fc(IgG) fusion proteins and expression as dimers of lectin-Fc(IgG) monomers linked by disulfide bonds. (**a**) PCR amplification of pFUSE-Dectin-1-Fc. Lane 1—molecular weight marker GeneRulerTM 100 pb DNA (Thermo Fisher Scientific); lane 2—p pFUSE-Dectin-1(IgG2a); lane 3—pFUSE-Dectin-1-Fc(IgG2b) and lane 4—negative control. (**b**) PCR amplification of pFUSE-WGA-Fc(IgG2a). Lane 1—molecular weight marker Ladder 100 pb (Ludwig Biotec); lane 2—pFUSE-WGA-Fc(IgG2a) and lane 3—negative control. (**c**) Endonuclease digestion of pFUSE-lectin-Fc(IgG) plasmids: lane 1—molecular weight marker GeneRulerTM 100 pb DNA (Thermo Fisher Scientific); lane 2—pFUSE-Dectin-1-Fc(IgG2a); lane 3—pFUSE-Dectin-1-Fc(IgG2b); lane 4—pFUSE-WGA-Fc(IgG2a) and lane 5—GeneRulerTM Express DNA (Thermo Fisher Scientific). (**d**) Immunoblots of lectin-Fc(IgG) fusion proteins Dectin-1-Fc(IgG2a), Dectin-1-Fc(IgG2b) and wheat germ agglutinin(WGA)-Fc(IgG2a) showing the monomers of the lectin-Fc(IgG) fusion proteins of ~50 KDa under reducing conditions in comparison to the dimers of lectin-Fc(IgG) of ~100 KDa observed under non-reducing conditions. The molecular marker—Kaleidoscope™ (Bio-Rad).

PCR amplicons for Dectin-1 and WGA were digested with endonucleases as described and cloned into the pFUSE plasmids. Cloning of Dectin-1 and WGA coding sequences were confirmed by PCR using the ligated plasmids as templates. Plasmids pFUSE-Dectin-1-Fc(IgG2a) and pFUSE-Dectin-1-(IgG2b) rendered amplicons of 539 bp (Figure 1a) whereas pFUSE-WGA-Fc(IgG2a) resulted in an amplicon of 545 bp (Figure 1b). Endonuclease digestion with *EcoRI/NheI* confirmed the lectin insertions into plasmids (lectins + Fc regions, with 1249, 1278 and 1257 bp fragments generated, respectively (Figure 1c). Upon expression and purification, immunoblot analysis of purified lectin-Fc(IgG) proteins using an anti-mouse IgG-AP conjugate revealed bands of approximately 50 KDa in the reducing condition, corresponding to the molecular weight of lectin-Fc(IgG) monomers (Figure 1d). In the absence of β-mercaptoethanol, a band of approximately 100 KDa was observed, corresponding to the molecular weight of the dimerized proteins composed of the two identical lectin-Fc(IgG) monomers linked by disulfide bonds. Weak bands of 50 KDa under non-denaturing conditions for the three fusion proteins were probably the detection of trace amounts of lectin-Fc(IgG) monomers.

### 3.2. Lectin-Fc(IgG) Proteins Binding to A. fumigatus Germinating Conidia

**Figure 2:**
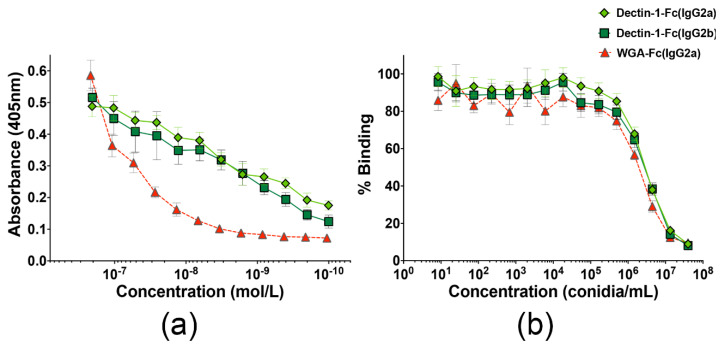
Lectin-Fc(IgG) fusion proteins binding to *Aspergillus fumigatus* 2 h germinating conidia. (**a**) The binding of the lectin-Fc(IgG) fusion proteins to the surface of the *A. fumigatus* conidia was initially evaluated by indirect enzyme-linked immunosorbent assay (ELISA) and demonstrated a dose-dependent binding. (**b**) Inhibition ELISA using *A. fumigatus* conidia demonstrated the specific binding of the lectin-Fc(IgG) proteins Dectin-1-Fc(IgG2a and IgG2b) and WGA-Fc(IgG2a) to the β-1,3-glucan and chitin molecules present on the surface of *A. fumigatus*, respectively.

*A. fumigatus* 2 h germinating conidia were used to coat 96-well microplates, as a source of β-1,3-glucan or chitin. Indirect ELISA demonstrated a dose-dependent binding of the three lectin-Fc(IgG) to conidia (Figure 2a). An irrelevant murine mAb (anti-CD4) was used as negative control and displayed no binging to fungal cells. At the highest protein concentration, all three proteins displayed similar binding intensity (*p* > 0.05); however, the Dectin-1-Fc(IgG2a) and Dectin-1-Fc(IgG2b) had higher binding values upon dilutions, and therefore lower Kds (respectively 2.6 × 10^−9^ and 8.4 × 10^−10^; *p* > 0.05), when compared to the WGA-Fc(IgG2a) control recognizing chitooligomers (Kd 1.7 × 10^−8^; *p* < 0.05, for both Dectin-1-Fc vs. WGA-Fc). An inhibition ELISA was also performed to verify whether the lectin-Fc(IgG) proteins Dectin-1-Fc(IgG2a), Dectin-1-Fc(IgG2b) or WGA-Fc(IgG2a) specifically bound the β-1,3-glucan or chitin molecules present in the cell wall of *A. fumigatus* germinating conidia, respectively (Figure 2b). Pre-incubation of the lectin-Fc(IgG)(s) with concentrations higher than 5.5 × 10^4^ *A. fumigatus* conidia/mL decreased the subsequent binding of these proteins to laminarin or chitooligomers in a dose-dependent manner, with 50% inhibition for 3.5 × 10^6^ conidia/well for both Dectin-1-Fc(IgG2a) and Dectin-1-Fc(IgG2b), and 2.4 × 10^6^ conidia/well for the WGA-Fc(IgG2a). Overall, the data suggest a specific binding of lectin-Fc(IgG) proteins to these target polysaccharides on *A. fumigatus* germinating conidia.

### 3.3. Lectin-Fc(IgG) Binding Pattern to A. fumigatus Germinating Conidia

**Figure 3:**
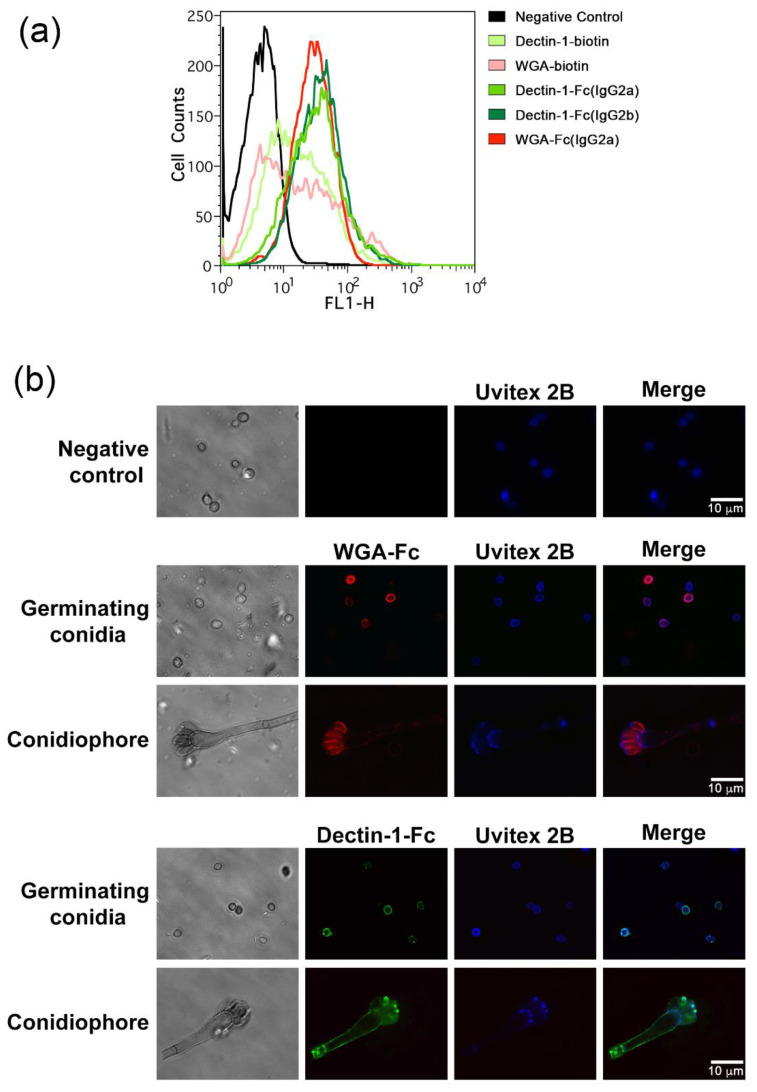
Binding pattern of lectin-Fc(IgG) fusion proteins to *A. fumigatus* conidia. (**a**) Flow cytometry and (**b**) Immunofluorescence analysis of *A. fumigatus* 2 h germinating conidia confirmed that the lectin-Fc(IgG) fusion proteins are capable of binding to the fungal surface, showing different distribution patterns. WGA-Fc (IgG2a) bound to the surface of conidia varying from a punctuated to a ring pattern, whereas no binding to hyphae was detected. In turn, Dectin-1-Fc(IgG) bound in a punctate pattern to both *A. fumigatus* hyphae and conidia. At least 20 fields were analyzed. Scale bar = 10 μm, for all panels.

Immunofluorescences were also performed to quantitatively determine and qualitatively analyze the pattern of binding of the lectin-Fc(IgG) fusion proteins to their targets present on the cell wall of *A. fumigatus*. Initially, the labeled 2 h germinating conidia were analyzed by flow cytometry. Conidia incubated with either lectin-Fc(IgG) displayed higher fluorescence intensity than its respective control of lectin (native Dectin-1 or WGA, *p* < 0.05; Figure 3a). Conidia were further labeled with chitin-binding Uvitex 2B for cell wall tracking and the labeling pattern of lectin-Fc(IgG) proteins were analyzed by microscopy (Figure 3b). Upon incubation, germinating conidia displayed strong binding with WGA-Fc(IgG2a), varying from a continuous ring-shaped to a gear pattern, with some bright dotted regions and broad variation of fluorescence intensity among conidia, along with a strong binding to phialides; in contrast, WGA-Fc(IgG2a) bound weakly to vesicles and hyphae in the conidiophores. The Dectin-1-Fc(IgG) showed a bright dotted fluorescent pattern on hyphae and at the tip of phialides, and also some punctuated regions of random distribution on the germinating conidia. The mainly punctuated distribution on the cell wall of phialides for both lectin-Fc(IgG) possibly indicates scars resulting from the conidiogenesis process.

### 3.4. Lectin-Fc(IgG) Proteins Show Enhanced Binding during Differentiation of A. fumigatus Conidia

The intensity and binding pattern of the lectin-Fc(IgG) fusion proteins to *A. fumigatus* conidia at different stages of germination were evaluated (resting 0 h, germinating conidia, 2 and 4 h and germinating conidia/hyphae, 8 h). Flow cytometry analysis showed that Dectin-1-Fc(IgG2a) bound weakly to resting conidia and efficiently to 2 h germinating conidia, similarly to Dectin-1-Fc(IgG2b), and followed in intensity by WGA-Fc(IgG2a) proteins. Both Dectin-1-Fc(IgG2a) and Dectin-1-Fc(IgG2b) fusion proteins displayed 1.5 and a 2.0-fold increase in binding to germinating conidia (4 h) and hyphae (8 h), respectively, when compared to 2 h germinating conidia (2 h, Supplementary Figure S1a). In turn, WGA-Fc(IgG2a) displayed an increase in binding intensity to 4 h germinating conidia (1.4 fold), whereas 8 h germinating conidia/hyphae had lower binding of WGA-Fc(IgG2a) compared to 2 h germinating conidia.

The binding pattern of the lectin-Fc(IG) proteins to *A. fumigatus* germinating conidia and hyphae were also analyzed (Supplementary Figure S1b). In all cases, the fusion proteins co-localized with the Uvitex 2B present on the cell surface. The lectin-Fc(IgG) bound 2 h germinating conidia in a punctuated manner, which coincided with the exposure of targets on the conidia surface, as observed in previous tests. Germinating conidia at 4 and 8 h displayed a much more intense binding for all lectin-Fc(IgG), whereas hyphae displayed a bright binding along their entire length for Dectin-1-Fc(IgGa) and Dectin-1-Fc(IgGb), and only at their tip for WGA-Fc(IgG2a). Additionally, the WGA-Fc(IgG2a) showed a weak binding along the hyphae, consistent with the results obtained by flow cytometry.

### 3.5. Lectin-Fc(IgG) Proteins Directly Inhibited A. fumigatus Germination

**Figure 4:**
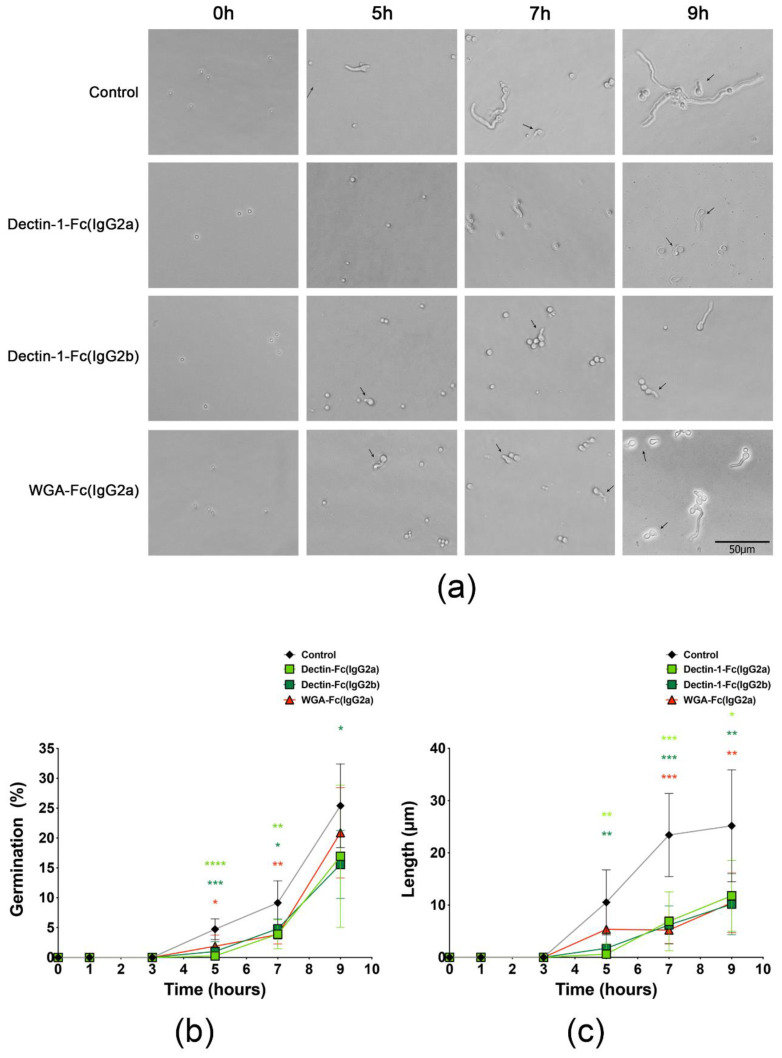
Pre-incubation with the lectin-Fc(IgG) altered the kinetics of conidia germination, hyphae formation and hyphal length. (**a**) Black arrows indicate the initial hyphal formation at 5 h, with rapid hyphae elongation observed at 7 h and 9 h in the control groups. The presence of lectin-Fc(IgG) was able to inhibit conidia germination and subsequent hyphal formation, resulting in shorter elements observed at 5 h, 7 h and 9 h. The experiments were performed in duplicate with at least three independent trials. Scale bar = 50 μm. (**b**,**c**) Quantitative evaluations by microscopy demonstrated that pre-incubation with the lectin-Fc(IgG) fusion proteins reduced (**b**) the percentage of germination events and (**c**) the hyphal length, with colors denoting the distinct groups compared to controls (* *p* < 0.05, ** *p* < 0.01, *** *p* < 0.001 and **** *p* < 0.0001). The experiments were performed in duplicate with at least three independent trials. Comparison among groups was performed by One-way ANOVA.

Incubation of *A. fumigatus* conidia with each of the lectin-Fc(IgG) fusion proteins led to a reduction of conidia germination events and a reduced overall length of the formed hyphae (Figure 4a). Inhibitory effects on germination were observed at time-points as early as 5 h, with inhibition values of 94.7% for Dectin-1-Fc(IgG2a), 78.8% for Dectin-1-Fc(IgG2b) and 60.3% for WGA-Fc(IgG2a) (Figure 4b). Inhibition effects were also significant at 7 h for the three fusion proteins evaluated (*p* < 0.05), but only the Dectin-1-Fc(IgG2b) displayed a statistically significant inhibition of hyphal formation at 9 h (38.7% inhibition, Figure 4b). Moreover, we also observed that the lectin-Fc(IgG) treatment slowed the hyphae elongation and decreased their length at time-points as early as 5 h (Figure 4c), with highest efficacy for the Dectin-1-Fc(IgG2a) (94.2% inhibition), followed by Dectin-1-Fc(IgG2b) (83.5%) and WGA-Fc(IgG2a) (49.1%). Upon 7 h and 9 h, hyphae lengths of treated groups were also smaller (Figure 4a), with similar inhibition efficacy values among the fusion proteins in the ranges of 70.5–77.6% and 53.2–59.6%, respectively (Figure 4c; *p* < 0.05).

### 3.6. Influence of Lectin-Fc(IgG) Proteins on A. fumigatus Biofilm Formation

The influence of lectin-Fc(IgG) proteins on biofilm formation was assessed by quantifying total biomass using 1% crystal violet (Supplementary Figure S2). The absorbance values recorded were proportional to the amount of biofilm biomass, which comprises hyphae and extracellular polymeric material. As a control, the addition of amphotericin B in the culture medium significantly abrogated fungal growth and biomass formation, thus validating the method used. Incubation with the lectin-Fc(IgG) fusion proteins demonstrated a dose-dependent inhibitory effect on biofilm formation. At 24 h, the Dectin-1-Fc(IgG2a) and Dectin-1-Fc(IgG2b) proteins had inhibitory effects only at 10 μg/mL, of about 10% inhibition, whereas the WGA-Fc(IgG2a) led to a 23% inhibition (Supplementary Figure S2a, *p* < 0.05). However at 48 h, inhibitory effects were more prominent, with inhibition values of 20% for the three fusion proteins at 10 μg/mL and about 14% for Dectin-1-Fc(IgG2b) and WGA-Fc(IgG2a) at 5 μg/mL (Supplementary Figure S2b, *p* < 0.05).

### 3.7. Lectin-Fc(IgG) Fusion Proteins Enhanced Complement Activation and A. fumigatus Growth Inhibition

**Figure 5:**
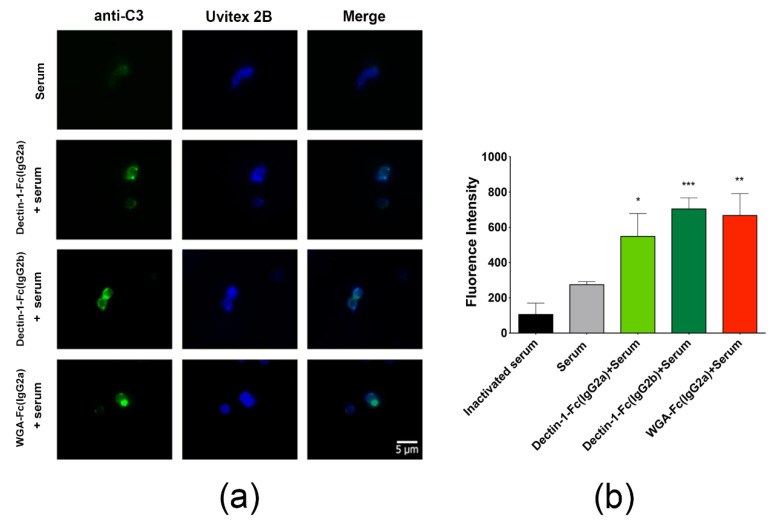
Lectin-Fc(IgG) binding enhanced complement activation resulting in *A. fumigatus* killing. (**a**,**b**) Opsonization of the *A. fumigatus* conidia with the lectin-Fc(IgG) proteins increased the deposition of C3 protein, in relation to the control groups incubated with active or inactivated mouse serum as evaluated by (**a**) fluorescence microscopy (100×) and (**b**) flow cytometry displaying the fluorescence intensity of individual cells (* *p* < 0.05, ** *p* < 0.01, *** *p* < 0.001, compared to serum alone). Comparison among groups was performed by One-way ANOVA.

Opsonization of germinating conidia with lectin-Fc(IgG) fusion proteins increased the deposition of C3 proteins on the conidial surface. Fluorescence microscopy images displayed a pattern of brighter dotted areas corresponding to a greater deposit of C3 protein on the surface of opsonized conidia, in comparison to the group incubated only with inactivated or active serum, which displayed no labeling and only a discrete surface labeling of bound C3 protein, respectively (Figure 5a). Quantification of fluorescence intensity by flow cytometry, demonstrated the highest values for Dectin-1-Fc(IgG2b), with an increase of 155.3% (*p* < 0.001), followed by a 141.9% for WGA-Fc(IgG2a) (*p* < 0.01) and 99.1% Dectin-1-Fc(IgG2a) (*p* < 0.05), in each case in comparison to fluorescence intensities of serum alone (Figure 5b). 

### 3.8. Opsonization with Lectin-Fc(IgG) Proteins Increased Phagocytosis of Germinating Conidia by Macrophages

**Figure 6:**
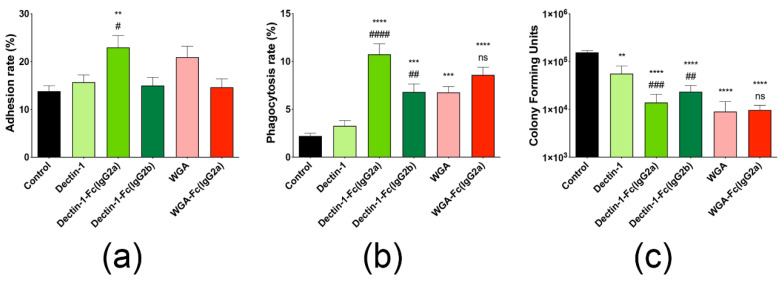
Opsonization with lectin-Fc(IgG) increased phagocytosis and fungal killing by alveolar macrophages. Graphical representation of the (**a**) adhesion and (**b**) phagocytosis rates for germinating conidia opsonized with PBS, Dectin-1, Dectin-1-Fc(IgG2a) or Dectin-1-Fc(IgG2b), WGA or WGA-Fc(IgG2a) prior to macrophage co-culture. The adhesion and phagocytosis rates were determined by the ratio of macrophages with attached or internalized fungi over the total number of macrophages. (**c**) The influence of lectin-Fc(IgG) on the conidia survival in the intraphagosomal environment of macrophages was also analyzed by CFU count. The assays were performed in duplicate and the results presented represent the average of at least three independent trials. Comparison among groups was performed by One-way ANOVA (** *p* < 0.01, *** *p* < 0.001 and **** *p* < 0.0001, in comparison to control treatment (PBS); # *p* < 0.05; ## *p* < 0.01 and ### *p* < 0.001 and #### *p* < 0.0001 in comparison to Dectin-1 treatment; ns—not significant, WGA-Fc(IgG2a) vs. WGA).

The interaction of *A. fumigatus* germinating conidia with macrophages upon opsonization with lectin-Fc(IgG) proteins was analyzed by dissecting the associations with macrophages into two main events, adhesion and phagocytosis. Pre-incubation of *A. fumigatus* conidia with the Fc-fusion protein Dectin-1-Fc(IgG2a) increased adhesion rates by 1.7 and 1.5 times in comparison to untreated and Dectin-1 controls (Figure 6a, *p* < 0.01 and *p* < 0.05, respectively). However, opsonization with Dectin-1-Fc(IgG2b) and WGA-Fc(IgG2a) had no significant effect on adhesion (Figure 6a). A higher impact was observed on the phagocytosis rates upon opsonization, with the Dectin-1-Fc(IgG2a) and Dectin-1-Fc(IgG2b) enhancing conidial internalization (5 times and 3 times, respectively), in comparison to the untreated control group (*p* < 0.0001 and *p* < 0.001, respectively, Figure 6b). In turn, WGA-Fc(IgG2a) showed a significant increase in the phagocytosis by about 4 and 1.3 times, respectively, in comparison to the untreated and native WGA controls (*p* < 0.001 and not significant, respectively).

### 3.9. Lectin-Fc(IgG) Proteins Enhance the Antifungal Efficacy of Macrophages

The effect of lectin-Fc(IgG) fusion proteins on the survival of conidia upon interaction with macrophage was assessed by determination of fungal viability by CFU (Figure 6c). Among the three fusion proteins evaluated, the WGA-Fc(IgG2a) seemed to induce better killing capacity by macrophages (94% growth inhibition, *p* < 0.0001), followed by the Dectin-1-Fc(IgG2a) and Dectin-1-Fc(IgG2b) fusion proteins (91% and 85%, respectively; *p* < 0.0001). The CFU values of the control groups incubated with native Dectin-1 and WGA proteins also showed a significant reduction with respect to the non-treated control group; however, only the Dectin-1-Fc(IgG2a) and Dectin-1-Fc(IgG2b) fusion proteins displayed a better relative capacity of inducing macrophage killing of internalized fungal structures than their respective lectin counterpart (*p* < 0.01).

### 3.10. Administration of Dectin-1-Fc(IgG2b) or WGA-Fc(IgG2a) Protected Mice against A. fumigatus Infection

**Figure 7:**
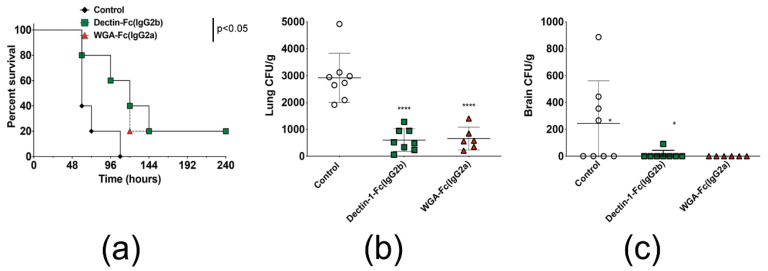
In vivo evaluation of the protective efficacy of Dectin-1-Fc(IgG2b) and WGA-Fc(IgG2a) fusion proteins. (**a**) Pre-treatment with Dectin-1-Fc(IgG2b) and WGA-Fc(IgG2a) had a slight protection in relation to the control group (100% mortality), where both lectin-Fc(IgG) proteins showed a survival rate of 20%, increasing the median survival by 2 days (*p* = 0.046). (**b**) Treatment with Dectin-1-Fc-(IgG2b) or WGA-Fc (IgG2a) reduced the fungal burden by 80% and 78%, respectively, in the lungs of the infected animals in comparison to the control group (**** *p* < 0.0001). (**c**) The fusion proteins also showed a trend in decreasing the spread of conidia within the host, inhibiting the fungal growth in the brain (* *p* = 0.05, in comparison to control).

The in vivo protective efficacy of lectin-Fc(IgG) fusion proteins was evaluated using a C57BL/6 mouse model of *A. fumigatus* infection. Immunosuppressed mice were intratracheally challenged with a lethal inoculum of *A. fumigatus* resting conidia after intraperitoneal administration of PBS or 10 μg of Dectin-1-Fc(IgG2b) or WGA-Fc(IgG2a), and the mice from different groups were observed daily for 30 days (Figure 7A). Animals began to die 60 h after the infection. However, 80% of the non-treated animals died within the first 3 days, reaching 100% death on day 5 post-infection, with a median survival of three days. The pre-treatment with both lectin-Fc(IgG) proteins offered a slight benefit for the animals, doubling the median survival to 5 days and allowing a 20% survival rate by the end of the study (*p* < 0.05). These surviving animals were euthanized at the end of the trial, and no CFUs were detected, suggesting a complete resolution of the infection. Animals were also infected with a sublethal inoculum of *A. fumigatus*, to further evaluate the protective effect of the lectin-Fc(IgG) fusion proteins. The fungal loads on different organs were analyzed 4 days after infection, and the values of each condition compared. Treatment with Dectin-1-Fc(IgG2b) and WGA-Fc(IgG2a) significantly reduced the pulmonary fungal load, in comparison to the control by 80% and 78%, respectively (Dectin-1-Fc(IgG2b) 5.98 × 10^2^ CFU/g vs. WGA-Fc(IgG2a) 6.57 × 10^2^ CFU/g vs. PBS 2.92 × 10^3^ CFU/g) (Figure 7B). Fungal load in the brain was detected in a single animal treated with Dectin-1-Fc(IgG2b) and was not detectable in WGA-Fc(IgG2a) treated mice, indicating a decreased dissemination to or a reduced fungal growth in this organ. In the untreated group it was possible to observe the presence of fungi in some animals, with average values of approximately 2.44 × 10^2^ CFUs/g (Figure 7C, *p* = 0.05). Finally, no fungal growth was observed in spleens of either condition.

## 4. Discussion

*A. fumigatus* is an opportunistic fungus that causes various respiratory illnesses and clinical manifestations in immunocompetent hosts, and severe invasive infections in immunosuppressed patients. In recent years, the overall incidence of invasive aspergillosis has increased from 200,000 to >300,000 cases, with an associated mortality ranging from 30 to 80% [11]. Emergence of azole and amphotericin B resistance at alarming rates and increase in clinical failure has been reported [61,62], and has been lately correlated to the use of antifungal in agriculture [63]. Altogether, new strategies for the therapeutic management of aspergillosis are urgently needed [64]. Passive immunization has been reconsidered as an alternative to the use of antifungals due to the lack of efficient and broad-spectrum therapeutic options for individuals in the risk groups for mycoses and the absence of resistance to antibody-based therapies [20].

Recent studies have suggested that natural carbohydrate ligands conjugated with the antibody-effector region are an alternative to the use of mAbs, in particular since certain carbohydrates lack an immunogenic potential. Our group [43,44] developed and characterized three lectin-Fc(IgG) proteins: Dectin-1-Fc(IgG2a), Dectin-1-Fc(IgG2b) and WGA-Fc(IgG2a). These fusion proteins are composed of the lectin binding domain, Dectin-1 or WGA, fused to the Fc portion of murine immunoglobulins, resulting in lectin-Fc(IgG) fusion proteins having affinity for β-1,3-glucan or chitin polysaccharides. These proteins recognize ubiquitous fungal cell wall carbohydrates and have emerged as important therapeutic options to protect the host against various fungal species without the need of a prior diagnosis, with already promising results against *C. albicans*, *C. neoformans* and *H. capsulatum* [43,44].

Biochemical characterization using immunobloting confirmed that the ~50 kDa lectin-Fc(IgG) monomers form dimers through disulfide bonds, where the resulting dimer should exhibit a greater avidity compared to a single monomer. The fusion of the immunoglobulins effector region did not affect lectin binding to the substrates, and Dectin-1-Fc(IgG2a) and Dectin-1-Fc(IgG2b) proteins showed specific binding to β-1,3-glucans present in the *A. fumigatus* germinating conidia cell wall, whereas the WGA-Fc(IgG2a) bound to the chitin present on this structure. These proteins also bind to the surface of *H. capsulatum, C. neoformans* and *C. albicans* [43,44] and have the potential to be used in broad-spectrum therapies.

An immunofluorescence assay was also performed to further characterize the recognition of polysaccharides by lectin-Fc(IgG) proteins. Both lectin-Fc(IgG) co-localized with Uvitex 2B on the surface of germinating conidia and displayed a fluorescent ring pattern with a mainly punctuated and specific distribution to certain points of the cell wall, possibly due to exposure of their target molecules resulting from the germination process. Despite co-localization, differences in labeling among samples treated with WGA-Fc(IgG2a) and Uvitex 2B was expected, due to the smaller size of the latter compared to the WGA-Fc(IgG2a) protein, which can lead to better penetration and subsequent more intense staining, besides the fact that Uvitex 2B could bind to total chitin, whereas WGA-Fc recognizes chitooligomers [43]. No significant difference among lectin-Fc(IgG) proteins was observed with respect to the intensity of fungal cell binding, although WGA-Fc(IgG2a) protein had the lowest values. The latter result could be explained by the innermost framework property of chitin, being less likely accessible than β-1,3-glucan [65].

Since germination and differentiation are key events during aspergillosis establishment, lectin-Fc(IgG) fusion proteins binding to cells at different growth stages was also analyzed. Lectin-Fc(IgG) proteins displayed greater binding to germinating conidia, especially at regions of germ tube growth and the tip of hyphae, similar to previous results [66]. Indeed, the reduced accessibility of *β*-1,3-glucan on resting conidia was previously reported by Luther et al. [45]. However, as conidia swell and germinate (in vivo and in vitro), there is a rapid and substantial reorganization of the conidial cell wall, where melanin and the rodlet layer disappear and the target molecules are exposed on the surface, increasing their availability [67]. The higher amounts of accessible β-1,3-glucan and chitin at these points can explain the higher lectin-Fc(IgG) binding. With continuous differentiation and hyphae formation, however, structural changes cause an accumulation of polysaccharides such as galactomannans and galactosaminogalactans in the outer layers of the cell wall, which can exert a steric impediment to the binding of lectin-Fc(IgG) proteins, specifically the access to chitin by WGA-Fc(IgG2a).

Although the exact effect of these lectin-Fc(IgG) fusion proteins on the cell wall structure is unknown, their binding to β-1,3-glucan and chitin could somehow affect or destabilize the bonds between the two molecules and impact the cellular homeostasis, potentially affecting fungal viability and growth. To analyze this inhibitory effect, the influence of lectin-Fc(IgG) fusion proteins on germination was evaluated, and a reduction in the percentage of germinating conidia and the hyphal length were observed. This inhibitory effect after 5 h of co-incubation may be directly correlated to the greater binding observed in the immunofluorescence analysis. The binding of β-1,3-glucan to chitin constitutes the central framework and approximately 30% of *A. fumigatus* cell wall [65]; therefore, the ligation of the lectin-Fc(IgG) fusion proteins may somehow impacted this bond and thereby caused a delay in the development of hyphae. Similar results were obtained using anti-β-1,3-glucan mAbs [30,43,68]. This mechanism is not specific for *A. fumigatus*, since WGA-Fc(IgG2a) and Dectin-1-Fc(IgG) proteins have similar effects against *C. albicans, H. capsulatum* and *C. neoformans*, suggesting that these lectin-Fc(IgG) proteins have a therapeutic “pan-antifungal” activity.

Biofilms contribute to *A. fumigatus* virulence, mainly during the development of invasive aspergillosis and pulmonary aspergilloma [69]. Thus, interference with its development is an attractive option for the development of new therapies. Incubation with lectin-Fc(IgG) fusion proteins reduced biofilm biomass formation in a dose-dependent fashion. The dismantling of extracellular matrix (ECM) or inhibition of the secretion of their components could facilitate the binding of more lectin-Fc(IgG) fusion proteins to resident cells or increase the penetration of antifungals. The potential synergism with current clinically used antifungals is being further explored by our group. Additionally, we are currently evaluating the action of these lectin-Fc(IgG) fusion proteins on pre-formed biofilms. However, the complex biofilm structure although being target for the binding of these lectin-Fc(IgG), could prevent their penetration into the biofilm itself by chemically sequester free-lectin-Fc(IgG) or their binding could cause antifungal repulsion due to electrostatic or hydrophobic hindrance [69] thereby increasing the antifungal resistance [4] and reducing the overall inhibitory effects of both therapeutical drugs.

Additional mechanisms by which lectin-Fc(IgG) proteins potentialize their antifungal activities were investigated. For instance, pre-incubation of *A. fumigatus* conidia with lectin-Fc(IgG) enhanced the deposition of mouse complement proteins onto the cell surface, similar to that observed for other fungi [44]. Analysis with anti-C3 mAbs showed dotted ring patterns with brighter areas, in agreement with regions of higher cell wall target polysaccharides exposure that exhibit a greater lectin-Fc(IgG) fusion proteins binding. Therefore, opsonization of *A. fumigatus* conidia with lectin-Fc(IgG) fusion proteins increases complement activation through the classical pathway [42,70,71].

In addition, the impact of lectin-Fc(IgG) on *A. fumigatus* interaction with macrophages was evaluated. Pathogen recognition is the first step in phagocytosis [72] and several immune system receptors such as lectins [73] play an important role in recognition of fungi by phagocytic cells. Dectin-1-Fc(IgG2a) significantly increased the percentage of macrophage surface-adhered conidia. Moreover, the opsonization considerably increased phagocytosis rates compared to control groups. This observation, as a proof of principle, confirms that lectin-Fc(IgG) fusion proteins bind to target molecules present on the conidia and the Fc effector region is recognized by the Fc receptors (FcR) present on the macrophage, which increases fungal internalization. Similar results were previously observed when lectin-Fc(IgG) proteins increased internalization of zymosan, *C. albicans*, *C. neoformans* and *H. capsulatum* [43,44,74]. In addition, all fusion proteins enhanced the antifungal activity of macrophages and reduced fungal survival in the intraphagosomal environment, in a similar way to previous studies [7,43,44,73,74]. These results altogether strongly suggested that lectin-Fc(IgG) fusion proteins could directly impact phagocytic cells function and disease outcome in favor of the host.

We then evaluate the antifungal efficacy in vivo. We have elected WGA-Fc(IgG2a) and Dectin-1-Fc(IgG2b) to perform this experiment, since the latter offered better results than Dectin-1-Fc(IgG2a) in the experiments described above. Prophylactic administration of Dectin-1-Fc(IgG2b) and WGA-Fc(IgG2a) proteins significantly protected profoundly immunosuppressed C57Bl/6 mice against *A. fumigatus* infection, slowing down mortality for about 2 days. A considerable reduction in lungs fungal burden of treated mice was also observed, which explains the 20% survival rate of lectin-Fc(IgG) treated mice. Spreading of infection to the brain was observed in untreated mice, resulting in symptoms such as tremors, circular movements and rolling [75]; however, fungal dissemination and the related manifestations were not observed in lectin-Fc(IgG) treated mice. Treatment with lectin-Fc(IgG) proteins had already shown protective effects against fungal pathogens such *as C. albicans, H. capsulatum*, *P. carinii*, and *A. fumigatus*, reducing fungal burden in several organs [7,43,44,74]. A more refined analysis and multivariable in vivo protection studies are currently being performed in our facilities, including distinct fungal inocula, dosage of lectin-Fc(IgG) fusion proteins, administration prior to or after infection and use of distinct mouse strain.

In general, the three lectin-Fc(IgG) proteins displayed good antifungal activity and quite a significant protective effect against *A. fumigatus*; demonstrating their potential as biopharmaceuticals in antifungal therapies. These lectin-Fc(IgG) fusion proteins bind to the polysaccharides present on the fungal cell wall, and interact with FcRs, resulting in a greater opsonizing effect, complement activation and enhanced macrophage effector activities, with the consequent reduction of the viability of internalized fungi. The recognition of antigens present in several pathogenic fungi allows the implementation of a universal antifungal strategy without the need for prior diagnosis and could be applied in various pharmaceutical formulations to treat the different presentations and clinical manifestations of mycoses. Additionally, binding to fungal cell wall could impair its polymerization, compromising its homeostasis and as a result, a less robust or loosen structure might allow a synergistic effect with clinically used antifungals. Future studies could be performed analyzing the influence of lectin-Fc(IgG) fusion proteins on other virulence factors, pharmacokinetic properties, and toxicity, as well as to develop new lectin-Fc(IgG) proteins with different target molecules to further increase the broad-spectrum potential of this approach.

## Figures and Tables

**Table 1:** Oligonucleotide sequences used in PCR amplification of lectin sequences.

PCR Oligonucleotides	
Dectin-1 primers	
Dectin-1 foward	5′-TTT GAA TTC GCA CAA TTC AGG GAG AAA TCC-3
Dectin-1 reverse	5′-TTT AGA TCT CAG TTC CTT CTC ACA GAT AC-3
WGA primers	
WGA forward	5′-TTT GAA TTC GCA GAG GTG CGG CGA GC-3
WGA reverse	5′-TTT CCA TGG CAG CGT CAC AGC CGC C-3

## Supplementary Materials

The following are available online at https://www.mdpi.com/2309-608X/6/4/250/s1, Figure S1: Binding of the lectin-Fc(IgG) proteins to the surface of the *A. fumigatus* conidia at different stages of growth, Figure S2: Biofilm formation inhibition by lectin-Fc(IgG) proteins.

## References

[1] Lamagni T.L., Evans B.G., Shigematsu M., Johnson E.M. (2001). Emerging trends in the epidemiology of invasive mycoses in England and Wales (1990-9). Epidemiol. Infect..

[2] van de Veerdonk F.L., Gresnigt M.S., Romani L., Netea M.G., Latge J.P. (2017). Aspergillus fumigatus morphology and dynamic host interactions. Nat. Rev. Microbiol..

[3] Denis B., Guiguet M., de Castro N., Mechai F., Revest M., Melica G., Costagliola D., Lortholary O. (2015). French Hospital Database on HIV National Agency for Research on AIDS and Viral Hepatitis, France CO4. Relevance of EORTC Criteria for the Diagnosis of Invasive Aspergillosis in HIV-Infected Patients, and Survival Trends Over a 20-Year Period in France. Clin. Infect. Dis..

[4] Abad A., Fernandez-Molina J.V., Bikandi J., Ramirez A., Margareto J., Sendino J., Hernando F.L., Ponton J., Garaizar J., Rementeria A. (2010). What makes Aspergillus fumigatus a successful pathogen? Genes and molecules involved in invasive aspergillosis. Rev. Iberoam. Micol..

[5] Low C.Y., Rotstein C. (2011). Emerging fungal infections in immunocompromised patients. F1000 Med. Rep..

[6] Jenks J.D., Spiess B., Buchheidt D., Hoenigl M. (2019). (New) Methods for Detection of Aspergillus fumigatus Resistance in Clinical Samples. Curr. Fungal Infect. Rep..

[7] Mattila P.E., Metz A.E., Rapaka R.R., Bauer L.D., Steele C. (2008). Dectin-1 Fc targeting of aspergillus fumigatus beta-glucans augments innate defense against invasive pulmonary aspergillosis. Antimicrob. Agents Chemother..

[8] Kontoyiannis D.P., Selleslag D., Mullane K., Cornely O.A., Hope W., Lortholary O., Croos-Dabrera R., Lademacher C., Engelhardt M., Patterson T.F. (2018). Impact of unresolved neutropenia in patients with neutropenia and invasive aspergillosis: A post hoc analysis of the SECURE trial. J. Antimicrob. Chemother..

[9] Procop G.W., Roberts G.D. (2004). Emerging fungal diseases: The importance of the host. Clin. Lab. Med..

[10] Goyal S., Castrillon-Betancur J.C., Klaile E., Slevogt H. (2018). The Interaction of Human Pathogenic Fungi With C-Type Lectin Receptors. Front. Immunol.

[11] Bongomin F., Gago S., Oladele R.O., Denning D.W. (2017). Global and Multi-National Prevalence of Fungal Diseases-Estimate Precision. J. Fungi.

[12] Shishodia S.K., Tiwari S., Shankar J. (2019). Resistance mechanism and proteins in Aspergillus species against antifungal agents. Mycology.

[13] Desoubeaux G., Cray C. (2018). Animal Models of Aspergillosis. Comp. Med..

[14] Taccone F.S., Van den Abeele A.M., Bulpa P., Misset B., Meersseman W., Cardoso T., Paiva J.A., Blasco-Navalpotro M., De Laere E., Dimopoulos G. (2015). Epidemiology of invasive aspergillosis in critically ill patients: Clinical presentation, underlying conditions, and outcomes. Crit. Care.

[15] Leal S.M., Cowden S., Hsia Y.C., Ghannoum M.A., Momany M., Pearlman E. (2010). Distinct roles for Dectin-1 and TLR4 in the pathogenesis of Aspergillus fumigatus keratitis. PLoS Pathog..

[16] Buil J.B., Hare R.K., Zwaan B.J., Arendrup M.C., Melchers W.J.G., Verweij P.E. (2019). The fading boundaries between patient and environmental routes of triazole resistance selection in Aspergillus fumigatus. PLoS Pathog..

[17] Nami S., Mohammadi R., Vakili M., Khezripour K., Mirzaei H., Morovati H. (2019). Fungal vaccines, mechanism of actions and immunology: A comprehensive review. Biomed. Pharmacother..

[18] Patterson T.F., Thompson G.R., Denning D.W., Fishman J.A., Hadley S., Herbrecht R., Kontoyiannis D.P., Marr K.A., Morrison V.A., Nguyen M.H. (2016). Practice Guidelines for the Diagnosis and Management of Aspergillosis: 2016 Update by the Infectious Diseases Society of America. Clin. Infect. Dis..

[19] Verweij P.E., Chowdhary A., Melchers W.J., Meis J.F. (2016). Azole Resistance in Aspergillus fumigatus: Can We Retain the Clinical Use of Mold-Active Antifungal Azoles?. Clin. Infect. Dis..

[20] Saylor C., Dadachova E., Casadevall A. (2009). Monoclonal antibody-based therapies for microbial diseases. Vaccine.

[21] Levitz S.M. (2017). Aspergillus vaccines: Hardly worth studying or worthy of hard study?. Med. Mycol..

[22] Vargas G., Honorato L., Guimaraes A.J., Rodrigues M.L., Reis F.C.G., Vale A.M., Ray A., Nosanchuk J.D., Nimrichter L. (2020). Protective effect of fungal extracellular vesicles against murine candidiasis. Cell Microbiol..

[23] Bryan R.A., Guimaraes A.J., Hopcraft S., Jiang Z., Bonilla K., Morgenstern A., Bruchertseifer F., Del Poeta M., Torosantucci A., Cassone A. (2012). Toward developing a universal treatment for fungal disease using radioimmunotherapy targeting common fungal antigens. Mycopathologia.

[24] Cassone A. (2008). Fungal vaccines: Real progress from real challenges. Lancet Infect. Dis..

[25] Sheppard D.C., Edwards J.E. (2004). Development of a vaccine for invasive aspergillosis. Clin. Infect. Dis..

[26] Pachl J., Svoboda P., Jacobs F., Vandewoude K., van der Hoven B., Spronk P., Masterson G., Malbrain M., Aoun M., Garbino J. (2006). A randomized, blinded, multicenter trial of lipid-associated amphotericin B alone versus in combination with an antibody-based inhibitor of heat shock protein 90 in patients with invasive candidiasis. Clin. Infect. Dis..

[27] Larsen R.A., Pappas P.G., Perfect J., Aberg J.A., Casadevall A., Cloud G.A., James R., Filler S., Dismukes W.E. (2005). Phase I evaluation of the safety and pharmacokinetics of murine-derived anticryptococcal antibody 18B7 in subjects with treated cryptococcal meningitis. Antimicrob. Agents Chemother..

[28] Moragues M.D., Omaetxebarria M.J., Elguezabal N., Sevilla M.J., Conti S., Polonelli L., Ponton J. (2003). A monoclonal antibody directed against a Candida albicans cell wall mannoprotein exerts three anti-C. albicans activities. Infect. Immun..

[29] Torosantucci A., Bromuro C., Chiani P., De Bernardis F., Berti F., Galli C., Norelli F., Bellucci C., Polonelli L., Costantino P. (2005). A novel glyco-conjugate vaccine against fungal pathogens. J. Exp. Med..

[30] Matveev A.L., Krylov V.B., Khlusevich Y.A., Baykov I.K., Yashunsky D.V., Emelyanova L.A., Tsvetkov Y.E., Karelin A.A., Bardashova A.V., Wong S.S.W. (2019). Novel mouse monoclonal antibodies specifically recognizing beta-(1-->3)-D-glucan antigen. PLoS ONE.

[31] Guimaraes A.J., Frases S., Gomez F.J., Zancope-Oliveira R.M., Nosanchuk J.D. (2009). Monoclonal antibodies to heat shock protein 60 alter the pathogenesis of Histoplasma capsulatum. Infect. Immun..

[32] Guimaraes A.J., Frases S., Pontes B., de Cerqueira M.D., Rodrigues M.L., Viana N.B., Nimrichter L., Nosanchuk J.D. (2011). Agglutination of Histoplasma capsulatum by IgG monoclonal antibodies against Hsp60 impacts macrophage effector functions. Infect. Immun..

[33] Nosanchuk J.D., Steenbergen J.N., Shi L., Deepe G.S., Casadevall A. (2003). Antibodies to a cell surface histone-like protein protect against Histoplasma capsulatum. J. Clin. Investig..

[34] Boniche C., Rossi S.A., Kischkel B., Barbalho F.V., Moura A.N.D., Nosanchuk J.D., Travassos L.R., Taborda C.P. (2020). Immunotherapy against Systemic Fungal Infections Based on Monoclonal Antibodies. J. Fungi.

[35] Casadevall A., Pirofski L.A. (2007). Antibody-mediated protection through cross-reactivity introduces a fungal heresy into immunological dogma. Infect. Immun..

[36] Guimaraes A.J., de Cerqueira M.D., Nosanchuk J.D. (2011). Surface architecture of histoplasma capsulatum. Front. Microbiol..

[37] Guimaraes A.J., Nakayasu E.S., Sobreira T.J., Cordero R.J., Nimrichter L., Almeida I.C., Nosanchuk J.D. (2011). Histoplasma capsulatum heat-shock 60 orchestrates the adaptation of the fungus to temperature stress. PLoS ONE.

[38] Gow N.A.R., Latge J.P., Munro C.A. (2017). The Fungal Cell Wall: Structure, Biosynthesis, and Function. Microbiol. Spectr..

[39] Hopke A., Brown A.J.P., Hall R.A., Wheeler R.T. (2018). Dynamic Fungal Cell Wall Architecture in Stress Adaptation and Immune Evasion. Trends Microbiol..

[40] Allen A.K., Neuberger A., Sharon N. (1973). The purification, composition and specificity of wheat-germ agglutinin. Biochem. J..

[41] Herre J., Gordon S., Brown G.D. (2004). Dectin-1 and its role in the recognition of beta-glucans by macrophages. Mol. Immunol..

[42] Czajkowsky D.M., Hu J., Shao Z., Pleass R.J. (2012). Fc-fusion proteins: New developments and future perspectives. EMBO Mol. Med..

[43] Liedke S.C. (2018). Proteínas Fc de Fusão Contra Glicanas da Parede Celular Fúngica: Construção e Avaliação das suas Propriedades Antifúngicas. Ph.D. Thesis.

[44] Liedke S.C., Miranda D.Z., Gomes K.X., Goncalves J.L.S., Frases S., Nosanchuk J.D., Rodrigues M.L., Nimrichter L., Peralta J.M., Guimaraes A.J. (2017). Characterization of the antifungal functions of a WGA-Fc (IgG2a) fusion protein binding to cell wall chitin oligomers. Sci. Rep..

[45] Luther K., Torosantucci A., Brakhage A.A., Heesemann J., Ebel F. (2007). Phagocytosis of Aspergillus fumigatus conidia by murine macrophages involves recognition by the dectin-1 beta-glucan receptor and Toll-like receptor 2. Cell Microbiol..

[46] Steele C., Rapaka R.R., Metz A., Pop S.M., Williams D.L., Gordon S., Kolls J.K., Brown G.D. (2005). The beta-glucan receptor dectin-1 recognizes specific morphologies of Aspergillus fumigatus. PLoS Pathog..

[47] Torosantucci A., Chiani P., Bromuro C., De Bernardis F., Palma A.S., Liu Y., Mignogna G., Maras B., Colone M., Stringaro A. (2009). Protection by anti-beta-glucan antibodies is associated with restricted beta-1,3 glucan binding specificity and inhibition of fungal growth and adherence. PLoS ONE.

[48] Englen M.D., Valdez Y.E., Lehnert N.M., Lehnert B.E. (1995). Granulocyte/macrophage colony-stimulating factor is expressed and secreted in cultures of murine L929 cells. J. Immunol. Methods.

[49] Trouplin V., Boucherit N., Gorvel L., Conti F., Mottola G., Ghigo E. (2013). Bone marrow-derived macrophage production. J. Vis. Exp..

[50] Laemmli U.K. (1970). Cleavage of structural proteins during the assembly of the head of bacteriophage T4. Nature.

[51] Guimarães A.J., Frases S., Cordero R.J., Nimrichter L., Casadevall A., Nosanchuk J.D. (2010). Cryptococcus neoformans responds to mannitol by increasing capsule size in vitro and in vivo. Cell Microbiol..

[52] Kaur S., Singh S. (2014). Biofilm formation by Aspergillus fumigatus. Med. Mycol..

[53] Mowat E., Butcher J., Lang S., Williams C., Ramage G. (2007). Development of a simple model for studying the effects of antifungal agents on multicellular communities of Aspergillus fumigatus. J. Med. Microbiol..

[54] Pierce C.G., Uppuluri P., Tristan A.R., Wormley F.L., Mowat E., Ramage G., Lopez-Ribot J.L. (2008). A simple and reproducible 96-well plate-based method for the formation of fungal biofilms and its application to antifungal susceptibility testing. Nat. Protoc..

[55] van de Sande W.W., Tavakol M., van Vianen W., Bakker-Woudenberg I.A. (2010). The effects of antifungal agents to conidial and hyphal forms of Aspergillus fumigatus. Med. Mycol..

[56] Chaieb K., Chehab O., Zmantar T., Rouabhia M., Mahdouani K., Bakhrouf A. (2007). In vitro effect of pH and ethanol on biofilm formation by clinical ica-positive Staphylococcus epidermidis strains. Ann. Microbiol..

[57] Guimarães A.J., de Cerqueira M.D., Zamith-Miranda D., Lopez P.H., Rodrigues M.L., Pontes B., Viana N.B., DeLeon-Rodriguez C.M., Rossi D.C.P., Casadevall A. (2019). Host membrane glycosphingolipids and lipid microdomains facilitate Histoplasma capsulatum internalisation by macrophages. Cell Microbiol..

[58] Cordero R.J., Liedke S.C., de S Araújo G.R., Martinez L.R., Nimrichter L., Frases S., Peralta J.M., Casadevall A., Rodrigues M.L., Nosanchuk J.D. (2016). Enhanced virulence of Histoplasma capsulatum through transfer and surface incorporation of glycans from Cryptococcus neoformans during co-infection. Sci. Rep..

[59] Ahlmann M., Hempel G. (2016). The effect of cyclophosphamide on the immune system: Implications for clinical cancer therapy. Cancer Chemother. Pharmacol..

[60] Silva R.L.H., Rosa-Milani E., Brunaldi M.O., Maffei C.M.L. (2019). Murine model of invasive pulmonary Aspergillosis: Follow-up of tissue injury, fungal burden and mortality with distinct elastase production strains. J. Mycol. Med..

[61] Perez-Cantero A., Lopez-Fernandez L., Guarro J., Capilla J. (2020). Azole resistance mechanisms in Aspergillus: Update and recent advances. Int. J. Antimicrob. Agents.

[62] Ashu E.E., Korfanty G.A., Samarasinghe H., Pum N., You M., Yamamura D., Xu J. (2018). Widespread amphotericin B-resistant strains of Aspergillus fumigatus in Hamilton, Canada. Infect. Drug Resist..

[63] Berger S., El Chazli Y., Babu A.F., Coste A.T. (2017). Azole Resistance in Aspergillus fumigatus: A Consequence of Antifungal Use in Agriculture?. Front. Microbiol..

[64] Romero M., Messina F., Marin E., Arechavala A., Depardo R., Walker L., Negroni R., Santiso G. (2019). Antifungal Resistance in Clinical Isolates of Aspergillus spp.: When Local Epidemiology Breaks the Norm. J. Fungi.

[65] Latge J.P., Mouyna I., Tekaia F., Beauvais A., Debeaupuis J.P., Nierman W. (2005). Specific molecular features in the organization and biosynthesis of the cell wall of Aspergillus fumigatus. Med. Mycol..

[66] Hohl T.M., Van Epps H.L., Rivera A., Morgan L.A., Chen P.L., Feldmesser M., Pamer E.G. (2005). Aspergillus fumigatus triggers inflammatory responses by stage-specific beta-glucan display. PLoS Pathog..

[67] Beauvais A., Latge J.P. (2015). Aspergillus Biofilm In Vitro and In Vivo. Microbiol. Spectr..

[68] Chaturvedi A.K., Kavishwar A., Shiva Keshava G.B., Shukla P.K. (2005). Monoclonal immunoglobulin G1 directed against Aspergillus fumigatus cell wall glycoprotein protects against experimental murine aspergillosis. Clin. Diagn. Lab. Immunol..

[69] Reichhardt C., Joubert L.M., Clemons K.V., Stevens D.A., Cegelski L. (2019). Integration of electron microscopy and solid-state NMR analysis for new views and compositional parameters of Aspergillus fumigatus biofilms. Med. Mycol..

[70] Dagenais T.R., Keller N.P. (2009). Pathogenesis of Aspergillus fumigatus in Invasive Aspergillosis. Clin. Microbiol. Rev..

[71] Noris M., Remuzzi G. (2013). Overview of complement activation and regulation. Semin. Nephrol..

[72] Nicola A.M., Casadevall A., Goldman D.L. (2008). Fungal killing by mammalian phagocytic cells. Curr. Opin. Microbiol..

[73] Gersuk G.M., Underhill D.M., Zhu L., Marr K.A. (2006). Dectin-1 and TLRs permit macrophages to distinguish between different Aspergillus fumigatus cellular states. J. Immunol..

[74] Rapaka R.R., Goetzman E.S., Zheng M., Vockley J., McKinley L., Kolls J.K., Steele C. (2007). Enhanced defense against Pneumocystis carinii mediated by a novel Dectin-1 receptor Fc fusion protein. J. Immunol..

[75] Zimmerli S., Knecht U., Leib S.L. (2007). A model of cerebral aspergillosis in non-immunosuppressed nursing rats. Acta Neuropathol..

