# Supplementary material for: Protective Efficacy of Lectin-Fc(IgG) Fusion Proteins In Vitro and in a Pulmonary Aspergillosis In Vivo Model
Source: J Fungi (Basel). 2020 Oct 27;6(4):250. doi: 10.3390/jof6040250 (PMC7712007; doi:10.3390/jof6040250)
Supplement: Supplementary file 1 [file jof-06-00250-s001.pdf]

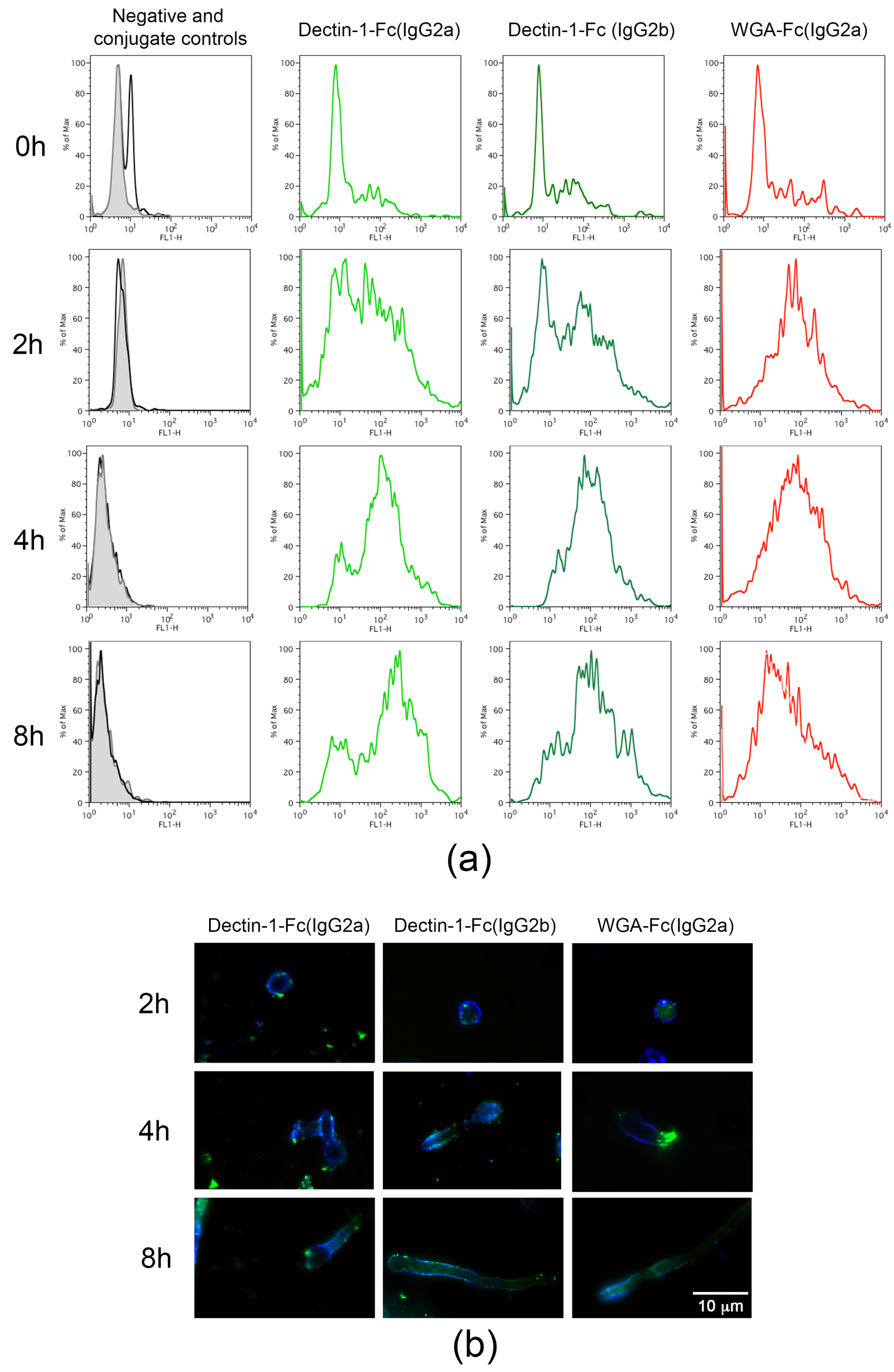

Supplementary Figure 1. The binding of the lectin-Fc(IgG) proteins to the surface of the *A. fumigatus* conidia at different stages of growth was evaluated by (**a**) flow cytometry and (**b**) Immunofluorescence microscopy (B), with at least 20 fields analyzed. Germinating conidia displayed high binding by Dectin-1-Fc(IgG2a) and Dectin-1-Fc(IgG2b) and wheat germ agglutinin(WGA)-Fc(IgG2a), whereas hyphae, had only higher binding by Dectin-1-Fc(IgG2a) and Dectin-1-Fc(IgG2b) in comparison to controls, accordance to results observed by immunofluorescence. Scale bar = 10 μm.

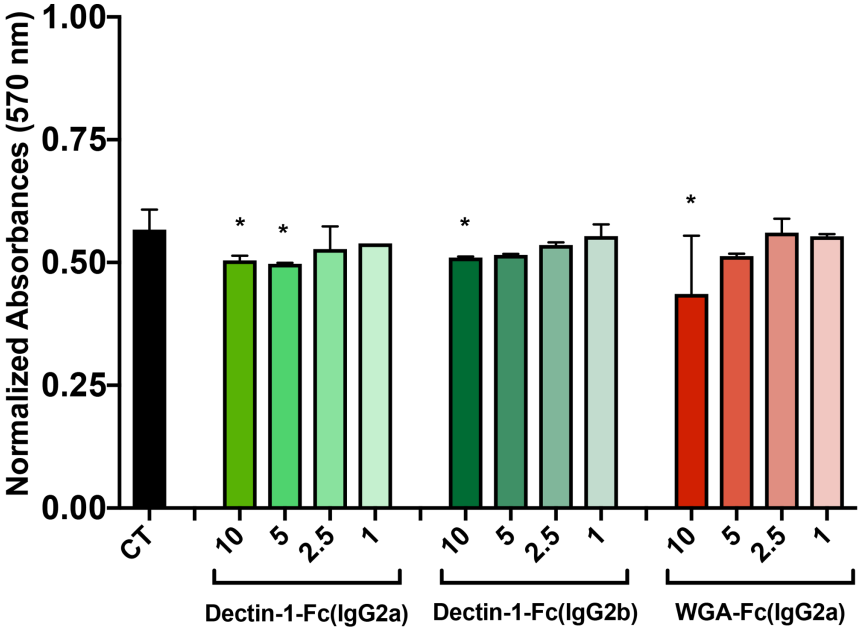

(a)

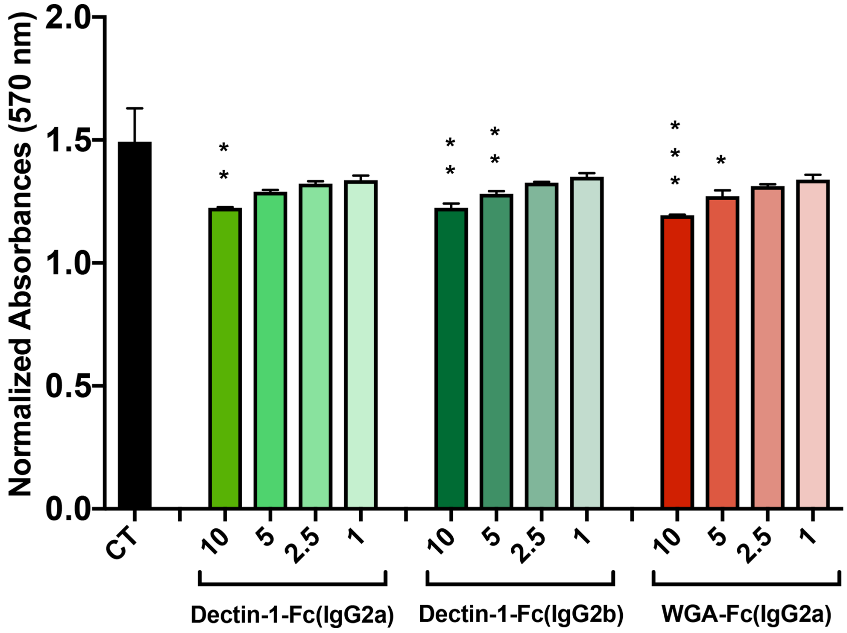

(b)

Supplementary Figure 2. Biofilm formation was also evaluated after (**a**) 24 and (**b**) 48 h incubation in the presence of PBS (control), Dectin-1-Fc(IgG2a), Dectin-1-Fc(IgG2b) and WGA-Fc(IgG2a) proteins. Amphotericin B or heat-killed conidia were used as negative controls and no signs of biofilm formation were observed. Lectin-Fc slightly decreased biofilm formation (~10%) only at the highest concentration in comparison to controls at 24 h biofilm inducing conditions. However, inhibition of biofilm formation was more expressive at 48 h, with about 20% inhibition at 10 μg/mL and 14% at 5 μg/mL. The experiments were performed in duplicate with at least three independent trials.
